# Abscisic Acid Promotes Jasmonic Acid Accumulation and Plays a Key Role in Citrus Canker Development

**DOI:** 10.3389/fpls.2019.01634

**Published:** 2019-12-20

**Authors:** Qin Long, Yu Xie, Yongrui He, Qiang Li, Xiuping Zou, Shanchun Chen

**Affiliations:** Citrus Research Institute, Southwest University/Chinese Academy of Agricultural Sciences, Chongqing, China

**Keywords:** citrus, bacterial canker, jasmonic acid, salicylic acid, abscisic acid, AOS1-2

## Abstract

Antagonism between jasmonic acid (JA) and salicylic acid (SA) plays pivotal roles in the fine-tuning of plant immunity against pathogen infection. In this study, we compared the phytohormonal responses to *Xanthomonas citri* subsp. *citri* (Xcc) between the citrus canker-susceptible (S) cultivar Wanjincheng orange (*Citrus sinensis* Osbeck) and -resistant (R) cultivar Jindan (*Fortunella crassifolia* Swingle). Upon Xcc infection, SA and JA were strongly induced in Jindan (R) and Wanjincheng orange (S), respectively, and JA appeared to contribute to citrus disease susceptibility by antagonizing SA-mediated effective defenses. A homologous gene encoding the allene oxide synthase (AOS) 1-2 enzyme, which catalyzes the first committed step in JA biosynthesis, was specifically upregulated in Wanjincheng orange (S) but not in Jindan (R). A promoter sequence analysis showed that abscisic acid (ABA)-responsive elements are enriched in the *AOS1-2* of Wanjincheng orange (S) but not in Jindan (R). Accordingly, ABA treatments could induce *AOS1-2* expression and JA accumulation, leading to enhanced citrus disease susceptibility in Wanjincheng orange (S), while the synthesis inhibitor sodium tungstate had the opposite effects. Moreover, ABA was specifically induced by Xcc infection in Wanjincheng orange (S) but not in Jindan (R). Thus, Xcc appeared to hijack host ABA biosynthesis to promote JA accumulation, which in turn suppressed effectual SA-mediated defenses to favor disease development in citrus. Our findings provide new insights into the molecular mechanisms underlying the differential citrus-canker resistance in citrus cultivars, and a new strategy for the biotechnological improvement of citrus canker resistance was discussed.

## Introduction

Citrus is the most economically important fruit-tree crop worldwide (Jia and Wang, 2014). Citrus canker, caused by *Xanthomonas citri* subsp. *citri* (Xcc), is a severe disease affecting most commercially grown citrus cultivars (Gottwald et al., 2002; Hao et al., 2016). It is a destructive disease of sweet orange, grapefruit, and tangelo. However, kumquat, satsuma mandarin, and ponkan are resistant to the disease (Koizumi, 1982; Song, 2002). Intensive studies of the differences in resistance-related mechanisms among different cultivars will provide a theoretical basis for citrus disease-resistance breeding and contribute to its targeted improvement.

A wide range of pathogenic microbes with different lifestyles and diverse infection strategies threaten the survival of plants. For defense, plants have developed sophisticated and flexible immune systems. Phytohormones, including salicylic acid (SA) and jasmonic acid (JA), play pivotal roles in the accurate regulation of plant immunity. In *Arabidopsis*, SA is crucial for immunity against pathogens having a biotrophic lifestyle, while JA mediates immunity against necrotrophic pathogens (Robert-Seilaniantz et al., 2011). Xcc has a biotrophic lifestyle (Gottig et al., 2010; Khalaf et al., 2011), which implies it is under the control of SA-mediated immunity. Consequently, exogenous treatments with SA or SA analogs reduce the incidence of citrus canker in susceptible cultivars (Francis et al., 2009; Graham and Myers, 2011; Wang and Liu, 2012). Over-expression of the *Arabidopsis NONEXPRESSOR OF PATHOGENESIS-RELATED 1* (*NPR1*) gene, acting downstream of SA, increases the resistance of susceptible cultivars to citrus canker (Zhang et al., 2010). A comparative analysis of the defense responses of different citrus genotypes to the Xcc-derived pathogen-associated molecular pattern flg22 showed that the expression of the SA-biosynthetic gene phenylalanine ammonia lyase 1 (*PAL1*) is significantly induced in resistant but not in susceptible citrus (Shi et al., 2015). These results underscore the importance of SA in citrus immunity against Xcc.

The SA pathway does not act independently within plants. Crosstalk with other phytohormonal pathways can promote or antagonize the SA pathway to regulate plant resistance (Audenaert et al., 2002; Grant and Jones, 2009; Robert-Seilaniantz et al., 2011). Antagonism between SA and JA allows plants to fine-tune immune responses by altering the hormonal concentrations or sensitivities (Pieterse et al., 2009; Zheng et al., 2012; Cui et al., 2018). However, pathogens have evolved the ability to manipulate plant hormone signaling to misdirect defense responses (Kazan and Lyons, 2014). For instance, some pathogens can activate the JA-signaling pathway to disable SA-mediated immune responses and, thus, facilitate pathogen proliferation (Brooks et al., 2005; Yang et al., 2017). Although the SA–JA crosstalk in defense is well characterized in non-tree plants, little is known regarding tree plants (Thaler et al., 2012; Islam et al., 2017).

Abscisic acid (ABA) plays important roles in developmental processes, abiotic stress resistance, and biotic stress responses (Walton, 1987; Audenaert et al., 2002; Mohr and Cahill, 2003). However, ABA has positive or negative effects on plant–microbe interactions, depending on the pathogen lifestyle (Robert-Seilaniantz et al., 2011). ABA can promote fungal (such as *Botrytis cinerea*) and bacterial (such as *Erwinia chrysanthemi* and *Pseudomonas syringae*) infections in tomato, while ABA pretreatments have conferred resistance to *Alternaria brassicicola* and *Plectosphaerella cucumerina* in *Arabidopsis* (Audenaert et al., 2002; Thaler and Bostock, 2004; Asselbergh et al., 2007; Ton et al., 2009). These studies indicate that there are different roles for ABA in necrotrophic and biotrophic infections. ABA also interacts with other phytohormones, such as JA and SA, during plant–pathogen interactions. Pathogen-modulated ABA signaling can rapidly antagonize SA-mediated defenses, and increased levels of ABA can increase the JA content and decrease the SA content (De Torres-Zabala et al., 2009; Fan et al., 2009). However, the role of ABA in the interactions between citrus and Xcc is unknown. Therefore, whether (and how) crosstalk between different phytohormone-signaling pathways regulates citrus canker resistance needs to be investigated.

In this study, after exposure to Xcc, a stronger SA pathway was induced in the resistant cultivar Jindan (*Fortunella crassifolia* Swingle), while a stronger JA pathway was induced in the susceptible cultivar Wanjincheng orange (*Citrus sinensis* Osbeck). We demonstrated that the high inducibility of ABA in susceptible Wanjincheng orange results in JA accumulation, possibly through the enhanced transcription of the ABA-responsive allene oxide synthase (*AOS*) *1-2* gene. The high level of JA in turn antagonizes effectual SA-mediated defenses to promote disease, while these effects were not found in the resistant Jindan.

## Materials and Methods

### Plant and Pathogen Materials

Wanjincheng orange (*C. sinensis* Osbeck) and Jindan (*F. crassifolia* Swingle) plants used for inoculation were grown in a glasshouse at temperatures ranging from 25 to 30°C at the National Citrus Germplasm Repository, Chongqing, China.

A type A strain of *X. citri* ssp. *citri*, Xcc was isolated from naturally infected sweet orange leaves from an orchard in Yunnan province, China.

### Assay of Resistance to Citrus Canker

The Xcc strain used for inoculation was provided as a bacterial suspension solution, which was diluted to the required concentration with double-distilled water. Using an optical density at 600 nm (OD600) of 0.5, which is equivalent to ~5 × 10^8^ colony-forming units (cfu) per mL, bacterial suspensions of 5 × 10^5^ cfu/mL were injected into the abaxial surfaces of citrus leaves using 5-mL needleless syringes or 1 µL of bacterial suspension was applied to each puncture site made with a pin (0.5 mm in diameter). Leaves were cultured in an incubator at 28°C, with 80% relative humidity and a 16-h/8-h light/dark photocycle. The *in vitro* assay for disease resistance was performed as described by Peng et al. (2017).

### Plant Hormone Determination

For SA: Leaves (0.5 g in fresh weight) were ground in liquid nitrogen and extracted in 10 mL of methanol using a 50-min ultrasonic treatment. After centrifugation at 3,000 rpm for 10 min, the supernatant was filtered through a 0.22-µm microporous membrane. The SA content was analyzed on a HPLC system (AB Sciex, USA) using a Hypersil column (4.5 × 200 mm, 5 μm, Thermo, USA) at a flow rate of 1.0 mL/min and column temperature of 30°C. Detection was monitored at 327 nm. The mobile phase was methanol:0.3% phosphoric acid (36:64 by volume). The injection volume was 10 µL. SA was determined using the external standard method (Riet et al., 2016).

For JA: Leaves (0.3 g in fresh weight) were ground in liquid nitrogen and extracted in 5 mL of 80% methanol containing 1% glacial acetic acid overnight at 4°C. After centrifugation at 3,000 rpm for 10 min, the supernatant was loaded sequentially onto ProElut PLS, ProElut PXC, and ProElut PWA columns (DiKMA, China) to purify JA as described previously (Tokuda et al., 2013). The JA content was analyzed on a HPLC system (AB Sciex) using an Eclipse XDB-C18 column (250 × 4.6 mm, 5 µm, Waters, Ireland) at 25°C. The mobile phase was methanol:acetic acid aqueous solution (pH 3.6; 1:1 by volume). The isocratic elution’s flow rate was 1 mL/min. The detection wavelength was 235 nm. The injection volume was 10 µL. JA was determined using the external standard method (Riet et al., 2016).

For ABA: Leaves (1.0 g in fresh weight) were ground in liquid nitrogen and extracted in 9 mL of 10 mM PBS buffer (pH 7.2). The supernatant was used to determine the ABA content following a plant enzyme-linked immunosorbent assay kit’s instruction (Mlbio, China).

### Plant Hormone and Inhibitor Treatments

SA, methyl jasmonate (MeJA), ABA, and sodium tungstate (ST) were purchased from Sigma (USA), and were diluted to the required 5-mM, 100-µM, 100-µM, and 5-mM concentrations, respectively (Eyre et al., 2006; Mohr and Cahill, 2007; Duan et al., 2015). Leaves were moistened with absorbent cotton wool pre-soaked with 5 mL of hormone, ST solution, or water after they had been injected with Xcc suspensions (5 × 10^5^ cfu/mL). For the ABA treatment alone, citrus leaves were injected with ABA solution or water using a 5-mL needleless syringe without Xcc infection. The treated leaves were cultured in an incubator at 28℃, with 80% relative humidity and a 16-h/8-h light/dark photocycle.

Effect of ST on bacterial viability was determined as follows: bacterial suspensions of the same concentration and volume were cultured with or without ST (5-mM), then OD600 values were detected at 4 and 8 h, respectively.

### RNA Extraction and Quantitative Real-Time RT-PCR

Total RNA was extracted from leaves using an EASY Spin Plant RNA Kit in accordance with the manufacturer’s instruction (Aidlab, China). A total of 0.5 to 1.0 µg RNA was the template for cDNA synthesis using a RevertAid First-Strand cDNA Synthesis Kit (Fermentas, Canada). The primers used for quantitative real-time PCR (qRT-PCR) were obtained from Primer-BLAST of NCBI (https://www.ncbi.nlm.nih.gov/tools/primer-blast/) and are listed in **Supplementary Table 1**. *CsActin* was used as the housekeeping gene reference. qRT-PCR was performed on a CFX96™ Real-Time System using iTaq™ universal SYBR Green Supermix (Bio-Rad, USA). The thermal cycling program consisted of an initial denaturation at 95°C for 3 min, followed by 40 cycles of 95°C for 15 s, 56°C for 30 s, and 72°C for 30 s. Primer specificity was verified using a melt-curve analysis. Data were analyzed using CFX Manager 3.1 software (Bio-Rad).

### Statistical Analysis

All the data were statistically analyzed by SPSS V20 software. Each value represented the averages ± standard deviations (SDs). The differences were evaluated using variance (ANOVA) based on Duncan’s multiple range test (*P* < 0.05) and significant differences were indicated by different letters, or using two-tailed Student’s *t*-test (*, *P* < 0.05; **, P < 0.01).

## Results

### Comparison of Canker Development in Wanjincheng Orange and Jindan

**Figure 1:**
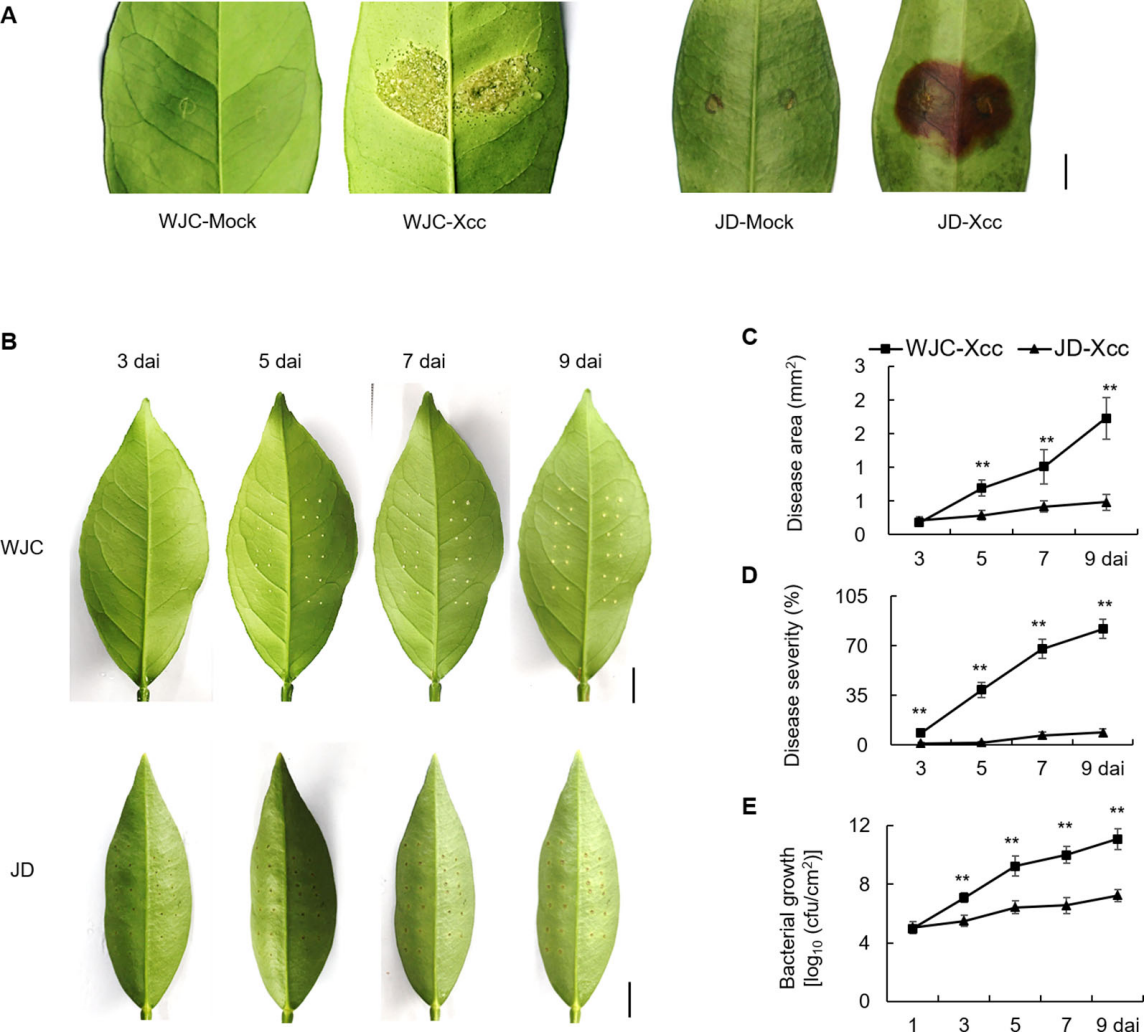
Canker disease resistance assay of Wanjincheng orange and Jindan. **(A)** Citrus canker symptoms on leaves of Wanjincheng orange and Jindan after *in vitro* inoculation with canker pathogen Xcc by injection. Scale bar = 0.5 cm. Fully expanded citrus leaves were injected with 5 × 10^5^ cfu/mL Xcc suspensions, and samples receiving a water treatment were used as blank controls. Photographs were taken at 10 days after treatment. **(B)** Citrus canker symptoms on different citrus leaves after inoculation with Xcc by pinprick. Scale bars = 1 cm. **(C)** Disease area on leaves of Wanjincheng orange and Jindan after inoculation. **(D)** Statistics of disease severity for Wanjincheng orange and Jindan leaves after inoculation. **(E)** Growth of Xcc in leaves of Wanjincheng orange and Jindan plants. Fully expanded citrus leaves were challenged with Xcc. 1 µL of bacterial suspension (5 × 10^5^ cfu/mL) was applied to each puncture site made with a pin (0.5 mm in diameter). Disease development and symptoms were observed every day after inoculation. The photographs were taken at 3, 5, 7, and 9 days after inoculation. Data represent the averages ± standard deviations (SDs) from three biological repeats, and nine independent leaves were observed for each replicate experiments. Statistical analyses were performed with two-tailed Student’s *t*-test to compare data from WJC-Xcc and JD-Xcc of the same time point (**, *P* < 0.01). WJC, Wanjincheng orange; JD, Jindan; Xcc, *Xanthomonas citri* ssp. *citri*; cfu, colony-forming units; dai, days after inoculation.

To identify differences of canker development between Wanjincheng orange (*C. sinensis* Osbeck) and Jindan (*F. crassifolia* Swingle), leaves of both species were inoculated with the Xcc suspension solution by two different methods, respectively. As shown in **Figure 1A**, typical canker symptoms of pustule were observed in Wanjincheng orange, while hypersensitive necrosis (blown region) occurred in Jindan at 10 days after inoculation (dai) by *in vitro* infiltration. To quantitatively evaluate disease resistance, the pinprick-inoculation method was used. Spongy and raised lesions were observed in Wanjincheng orange, whereas only weak water-soaking ring was detected in Jindan (**Figure 1B**). The statistical analysis showed that the disease area, disease severity and colony-forming units (cfu) in Wanjincheng orange were all significantly higher than that in the Jindan (**Figures 1C**–**E**). These data indicated that hypersensitive response in Jindan was stronger than that in Wanjincheng orange, and Jindan was remarkably resistant to Xcc compared with Wanjincheng orange.

### SA and JA Are Strongly Induced by Xcc in a Resistant and a Susceptible Citrus Cultivar, Respectively

**Figure 2:**
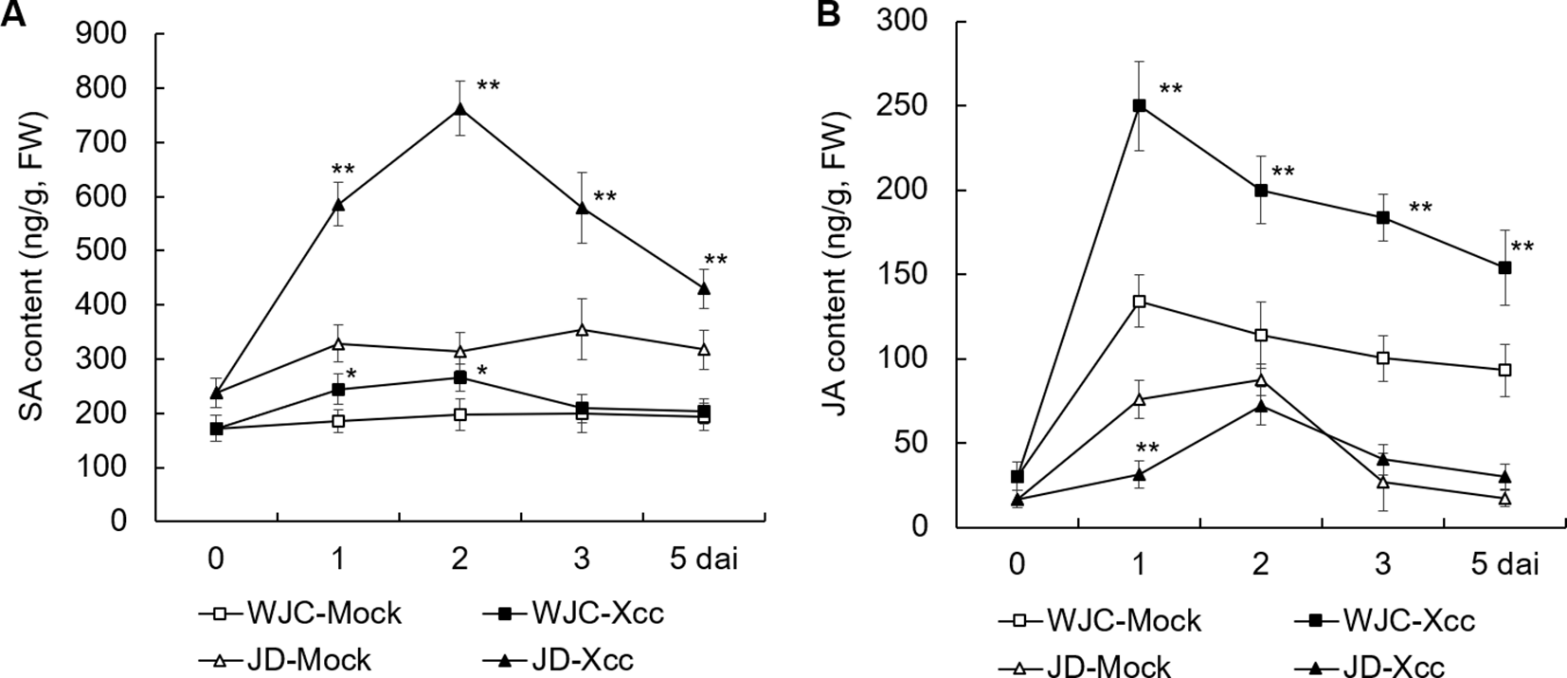
Dynamic changes in endogenous SA and JA contents of Xcc-resistant and -susceptible orange cultivars. **(A)** SA content of Wanjincheng orange and Jindan leaves. **(B)** JA content of Wanjincheng orange and Jindan leaves. Fully expanded citrus leaves were injected with 5 × 10^5^ cfu/mL Xcc suspensions, and samples receiving a water treatment were used as blank controls. SA and JA contents were determined by HPLC before and after inoculation. Error bars indicate the SDs of three biological repeats. Statistical significance related to their own mock control was determined by two-tailed Student’s *t*-test (*, *P* < 0.05; **, *P* < 0.01). WJC, Wanjincheng orange; JD, Jindan; Xcc, *Xanthomonas citri* ssp. *citri*; SA, salicylic acid; JA, jasmonic acid; dai, days after inoculation; FW, fresh weight.

To investigate the differences in the response of SA and JA to Xcc challenge between canker-susceptible (S) Wanjincheng orange and -resistant (R) Jindan, mature leaves of these two genotypes were inoculated with an Xcc suspension for 0, 1, 2, 3, and 5 d, and SA and JA levels were determined by HPLC. Before inoculation (0 d), the level of SA in Jindan (R) was about 40% higher than that in Wanjincheng orange (S). After Xcc inoculation, the contents of SA in both Wanjincheng orange (S) and Jindan (R) were increased compared with their respective mock treatment controls, and they peaked at 2 dai. Notably, the increase in the SA content in Jindan (R) was much greater than that in Wanjincheng orange (S). For example, the SA content in Jindan (R) was about 1.5 times higher than that of the control at 2 days after treatment with Xcc, and it was only about 30% higher in the Wanjincheng orange (S) (**Figure 2A**). Thus, SA was strongly upregulated by Xcc infection in resistant Jindan compared with susceptible Wanjincheng orange.

Furthermore, JA was strongly induced in the Wanjincheng orange (S) in both mock and Xcc treatments, with the latter resulting in a greater JA level compared with the former. For example, at 1 dai, the JA level peaked in Wanjincheng orange (S) at 249.8 and 134.2 µg/g in the Xcc-treated and mock samples, respectively, which were about 7.3 and 3.4 times higher than that of the 0-d counterparts, respectively. In contrast, the JA level was less induced in Jindan (R) mock and Xcc-treated samples (**Figure 2B**). Thus, JA was strongly upregulated by Xcc infection in susceptible Wanjincheng orange compared with in resistant Jindan.

### SA Synthesis Is Upregulated More in the Resistant Citrus, While JA Synthesis Is Upregulated More in the Susceptible Citrus

**Figure 3:**
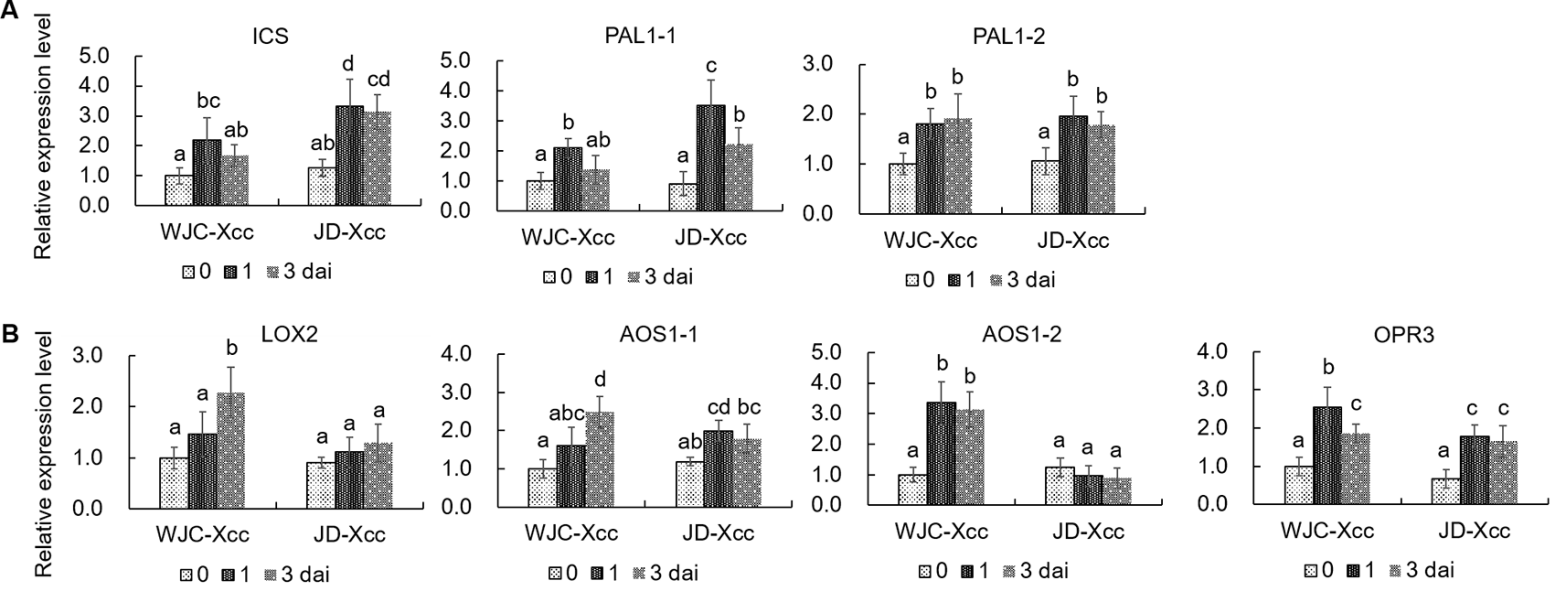
Transcriptional levels of genes involved in the synthesis of SA and JA. **(A)** Expression levels of genes involved in SA synthesis. **(B)** Transcriptional levels of genes involved in JA synthesis. Leaves from Wanjincheng orange (S) and Jindan (R) were injected with canker pathogen Xcc in solution. qRT-PCR was used to assess the expression levels of genes before and at 1 or 3 days after inoculation. Error bars represent the SDs of three biological replicates. Different letters above bars represent signiﬁcant differences from the 0 dai of WJC-Xcc based on Duncan’s multiple range test (*P* < 0.05). WJC, Wanjincheng orange; JD, Jindan; dai, days after inoculation; Xcc, *Xanthomonas citri* ssp. *citri*; ICS, isochorismate synthase; PAL, phenylalanine ammonia lyase; LOX, 13-lipoxygenases; AOS, 13-allene oxide synthase; OPR3, 12-oxophytodienoate reductase 3.

To unravel the underlying mechanisms accounting for the changes in SA and JA contents in the two varieties after inoculation, qRT-PCR was used to assess the expression levels of SA and JA synthesis-related genes. SA is synthesized through either the isochorismate synthase (ICS) or the PAL catalyzed step (Kumar et al., 2015). qRT-PCR results showed that the transcript levels of ICS, PAL1-1 and PAL1-2 were all upregulated in Wanjincheng orange (S) and Jindan (R) after Xcc inoculation. The increases in ICS and PAL1-1 were greater in Jindan (R) than in Wanjincheng orange (S), which was consistent with the higher level of SA in Jindan (R) (**Figure 3A**). Lipoxygenases (13-LOXs), 13-AOS, and 12-oxophytodienoate reductase 3 (OPR3) are important enzymes involved in JA synthesis (Turner et al., 2002; Toyoda et al., 2013; Poltronieri et al., 2015). The transcripts of these genes, including *LOX2*, *AOS1-1*, *AOS1-2*, and *OPR3*, were significantly increased in Wanjincheng orange (S) upon Xcc infection (**Figure 3B**), while transcript levels of *LOX2* and *AOS1-2* showed no increase in Jindan (R). Because AOS catalyzes the first committed step in the JA biosynthesis from hydroperoxides of α-linolenic acid (Mueller, 1997; Zdyb et al., 2018), the differential expression of the *AOS1-2* in Wanjincheng orange (S) and Jindan (R) suggested it plays a crucial role in the accumulation of JA in response to Xcc. The above results indicated that greater SA synthesis is induced in the resistant citrus, while greater JA synthesis is induced in the susceptible citrus after Xcc infection.

### A Stronger SA-Signaling Pathway Is Induced in the Resistant Citrus, While a Stronger JA-Signaling Pathway Is Induced in the Susceptible Citrus

**Figure 4:**
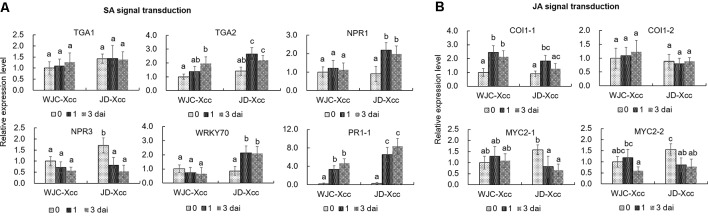
Expression levels of genes involved in SA and JA signal transduction. **(A)** Expression levels of genes involved in SA signal transduction. **(B)** Transcriptional levels of genes involved in JA signal transduction. Leaves from Wanjincheng orange (S) and Jindan (R) were inoculated with the canker pathogen Xcc by injection, and samples treated with water were used as blank controls. Expression levels of genes were assessed by qRT-PCR before and after inoculation. Error bars represent the SDs of three biological replicates. Different letters above bars represent signiﬁcant differences from the 0 dai of WJC-Xcc based on Duncan’s multiple range test (*P* < 0.05). WJC, Wanjincheng orange; JD, Jindan; Xcc, *Xanthomonas citri* ssp. *citri*; dai, days after inoculation; TGA, basic region/leucine zipper TGA transcription factors; NPR, non-expressor of PR proteins; WRKY, WRKY family of transcription factors; PR, pathogenesis-related proteins; COI1, coronatine insensitive 1; MYC2, basic helix–loop–helix MYC2 transcription factors.

The TGA family of basic region/leucine zipper transcription factors, NPR proteins, WRKY transcription factors and PR proteins are key members of the SA-signaling pathway that activate defense responses (Fan and Dong, 2002; Pieterse et al., 2009; Shi et al., 2013). qRT-PCR analyses showed that the transcripts of *TGA2*, but not *TGA1*, significantly increased at 1 dai in Jindan (R) but only slightly increased in Wanjincheng orange (S) at the same time. Furthermore, transcript levels of *NPR1* and *WRKY70* significantly increased in Jindan (R) upon Xcc infection, while no significant increases were observed in Wanjincheng orange (S). Moreover, *NPR3*, a negative regulator of SA-induced defense gene expression (Ding et al., 2018), was strongly suppressed in Jindan (R), but suppressed to a lesser degree in Wanjincheng orange (S). Like that of *TGA2*, the transcript level of *PR1* was strongly induced in Jindan (R), while to a lesser extent in Wanjincheng orange (S) (**Figure 4A**). Thus, the SA-signaling pathway was successfully and strongly induced in Jindan (R), but only moderately induced in Wanjincheng orange (S) upon Xcc infection, which mirrored the SA concentrations in the two cultivars.

Coronatine insensitive 1 (COI1) and MYC2-family basic helix–loop–helix transcription factors play important roles on the JA-signaling pathway (Wasternack and Hause, 2013). qRT-PCR analyses showed that these genes were upregulated in Wanjincheng orange (S), but less induced or downregulated in Jindan (R) after Xcc inoculation. For instance, the transcriptional levels of *COI1-1* at 1 dai and 3 dai in Wanjincheng orange (S) were about 1.5 and 1.2 times higher than the levels before inoculation, respectively, but they were less increased in Jindan (R) after Xcc infection. The transcripts of *MYC2-1* and *MYC2-2* were downregulated significantly in Jindan (R), while not in Wanjincheng orange (S) after inoculation (**Figure 4B**). These results indicated that the JA-signaling pathway was strongly induced in Wanjincheng orange (S) upon Xcc infection but less induced or suppressed in Jindan (R).

### JA Contributes to Canker Disease Susceptibility, Possibly Through Antagonizing SA-Mediated Effective Defense

**Figure 5:**
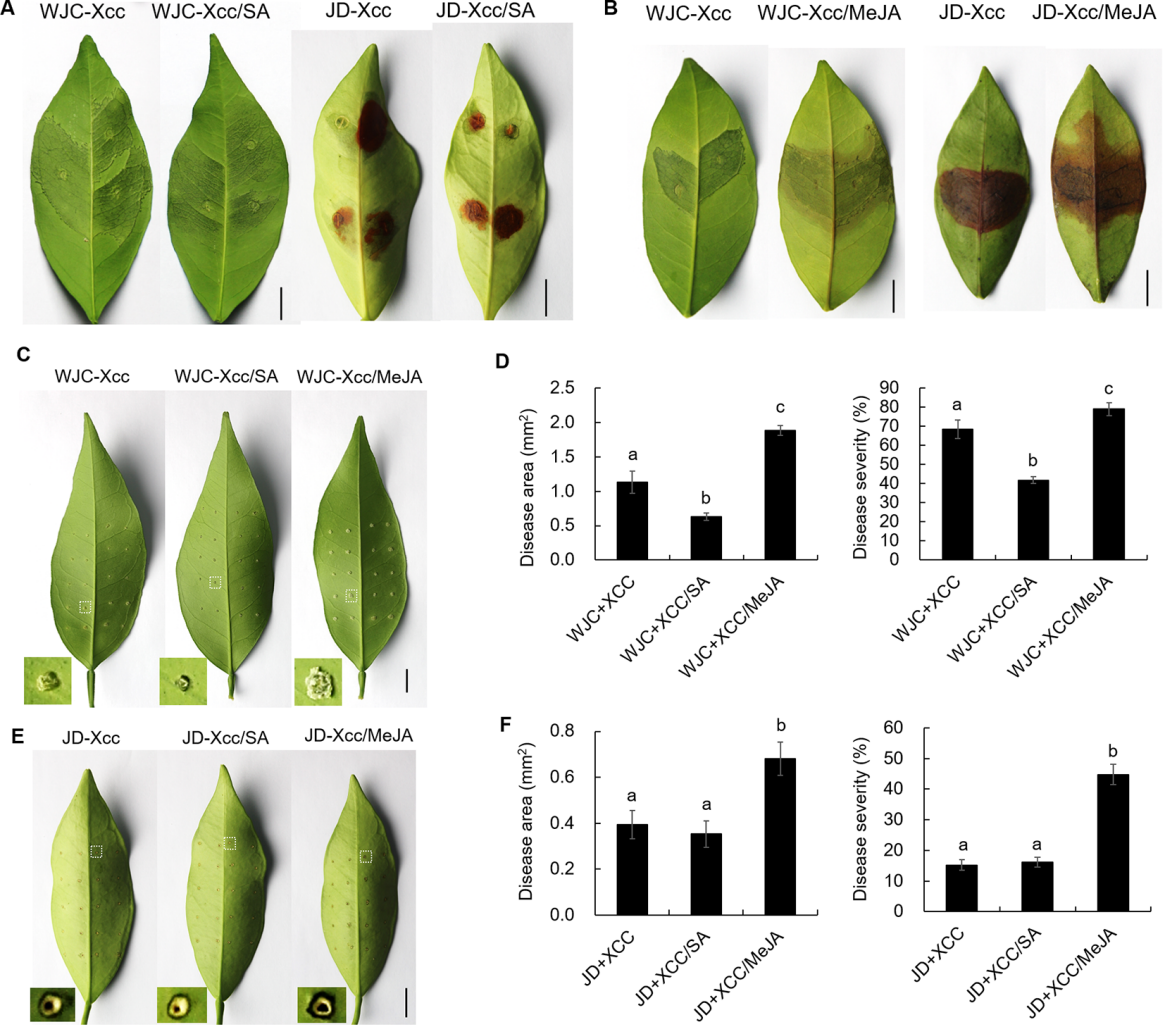
Effects of exogenous additions of SA and MeJA on Wanjincheng orange and Jindan leaves after infection with Xcc. **(A** and **B)** Effects of exogenous SA and MeJA on Wanjincheng orange and Jindan leaves after Xcc infection by injection. The photographs were taken at 8 days after inoculation. Scale bars = 1 cm. **(C)** Effects of exogenous SA and MeJA on Wanjincheng orange leaves after Xcc infection by pin-prick. The photographs were taken at 8 days after inoculation. Scale bars = 1 cm. **(D)** Disease area and disease severity levels for Wanjincheng orange leaves after inoculation and both SA and MeJA treatments. **(E)** Effects of exogenous SA and MeJA on Jindan leaves after Xcc infection by pinprick. The photographs were taken at 8 days after inoculation. Scale bars = 1 cm. **(F)** Statistics of diseased-spot squares and disease severity levels for Jindan leaves after inoculation and both SA and MeJA treatments. Data represent the averages ± SDs from three biological repeats, and nine independent leaves were observed for each replicate experiments. Different letters above bars represent signiﬁcant differences from the Xcc treatment based on Duncan’s multiple range test (*P* < 0.05). WJC, Wanjincheng orange; JD, Jindan; Xcc, *Xanthomonas citri* ssp. *citri*; SA, salicylic acid; MeJA, methyl jasmonate.

**Figure 6:**
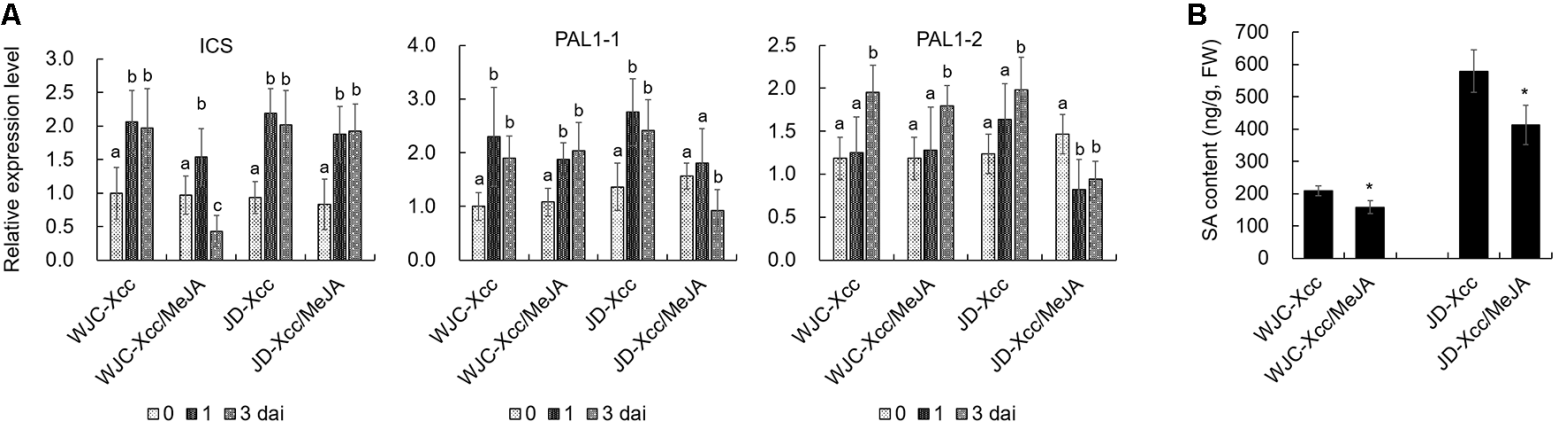
Effects of exogenous MeJA on SA synthesis in Wanjincheng orange and Jindan leaves after infection with Xcc. **(A)** Effects of MeJA on the expression of SA biosynthetic genes in Wanjincheng orange and Jindan leaves after infection with Xcc. Error bars represent the SDs of three biological replicates. Different letters above bars represent signiﬁcant differences from the 0 dai based on Duncan’s multiple range test (*P* < 0.05). **(B)** Effects of MeJA on the SA contents in Wanjincheng orange and Jindan leaves after infection with Xcc. Error bars indicate the standard deviations of three biological repeats. Statistical significance related to the control was determined by two-tailed Student’s *t*-test (*, *P* < 0.05). WJC, Wanjincheng orange; JD, Jindan; Xcc, *Xanthomonas citri* ssp. *citri*; SA, salicylic acid; MeJA, methyl jasmonate; ICS, isochorismate synthase; PAL, phenylalanine ammonia lyase.

The different variations in SA and JA levels in Wanjincheng orange (S) and Jindan (R) after Xcc infection suggested that they had different roles in citrus canker resistance. To confirm this, we treated Wanjincheng orange (S) and Jindan (R) with exogenous SA and MeJA (an active JA), respectively. Consistent with the SA level determination, the SA treatment significantly enhanced the resistance of the Wanjincheng orange (S) against Xcc introduced by *in vitro* infiltration or pinprick inoculation. For instance, the statistical analysis showed that the SA treatment significantly decreased the disease lesions and disease severity of Wanjincheng orange (S) in the pinprick inoculation experiments. In contrast, the MeJA treatment further aggravated the pustule and canker symptoms of Wanjincheng orange (S) and Jindan (R) upon Xcc infiltration (**Figures 5A**–**F**). Interestingly, the MeJA treatment promoted necrotic lesion formation that was associated with the hypersensitive response (HR) in Jindan (R), reminiscent of a report that MeJA promotes necrosis on leaves independent of SA synthesis in an HR-occurring cotton background (Sun et al., 2014). Moreover, the exogenous MeJA suppressed the expression levels of genes involved in SA synthesis after Xcc infection. In addition, SA levels in Wanjincheng orange (S) and Jindan (R) both decreased with MeJA treatment after inoculation (**Figures 6A**, **B**). Thus, JAs appear to contribute to canker disease susceptibility by antagonizing SA-mediated effective defenses.

### The ABA Accumulation Induced by Xcc Contributes to the High JA Level in Susceptible Citrus

**Figure 7:**
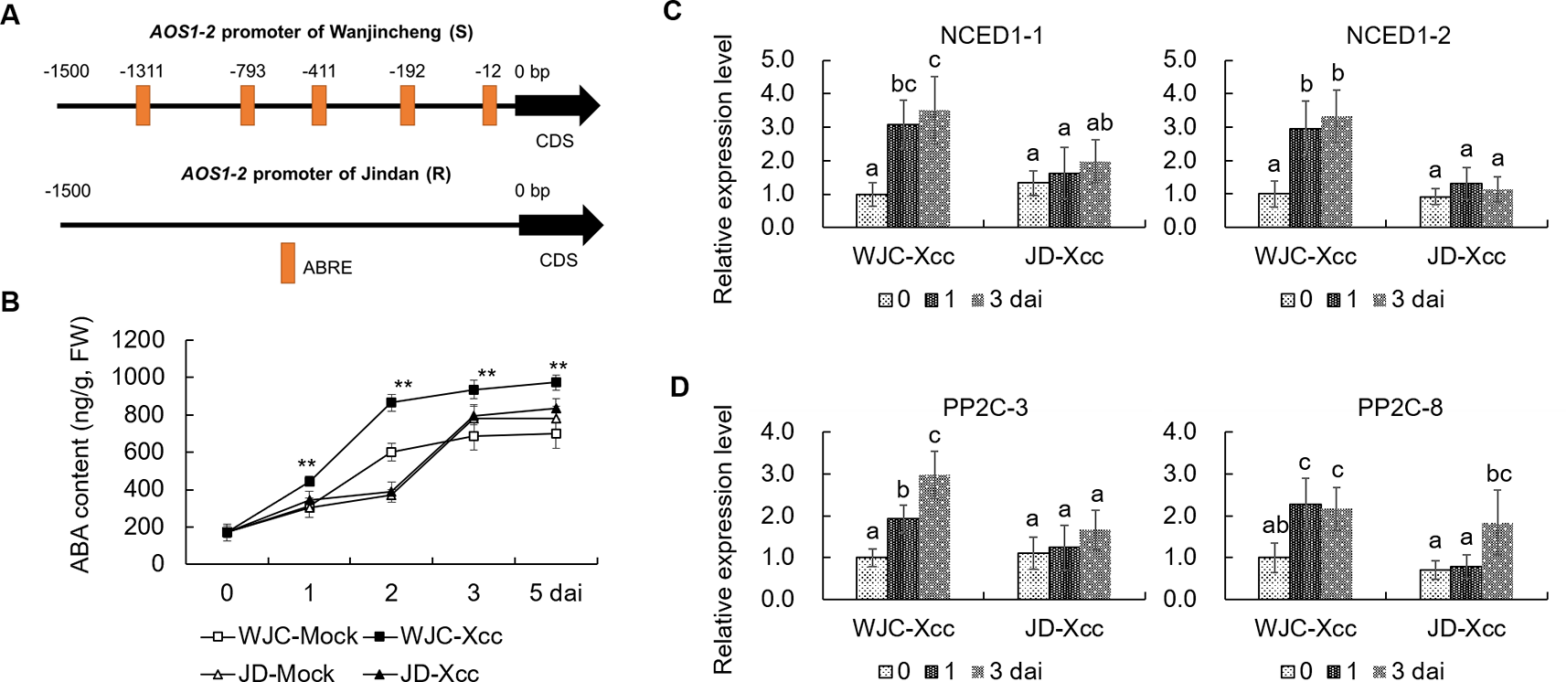
ABA was induced in Wanjincheng orange after infection with Xcc. **(A)** Cis-regulating motifs in the 1.5-kb promoters of *AOS1-2* genes analyzed using PlantCARE (http://bioinformatics.psb.ugent.be/webtools/plantcare/html/). **(B)** ABA content in Wanjincheng orange and Jindan leaves before and after Xcc inoculation. Error bars indicate the SDs of three biological repeats. Statistical significance related to their own mock control was determined by two-tailed Student’s *t*-test (**, *P* < 0.01). **(C)** Expression levels of genes involved in ABA synthesis. **(D)** Transcriptional levels of genes involved in ABA signal transduction. Fully expanded citrus leaves were injected with 5 × 10^5^-cfu/mL Xcc suspensions, and samples treated with water were used as blank controls. Error bars represent the SDs of three biological replicates. Different letters above bars represent signiﬁcant differences from the 0 dai of WJC-Xcc based on Duncan’s multiple range test (*P* < 0.05). WJC, Wanjincheng orange; JD, Jindan; AOS, 13-allene oxide synthase; ABRE, abscisic acid-responsive element (ACGTG); ABA, abscisic acid; dai, days after inoculation; FW, fresh weight; NCED1, 9-cis-epoxycarotenoid dioxygenase; PP2C, type 2C protein phosphatase.

**Figure 8:**
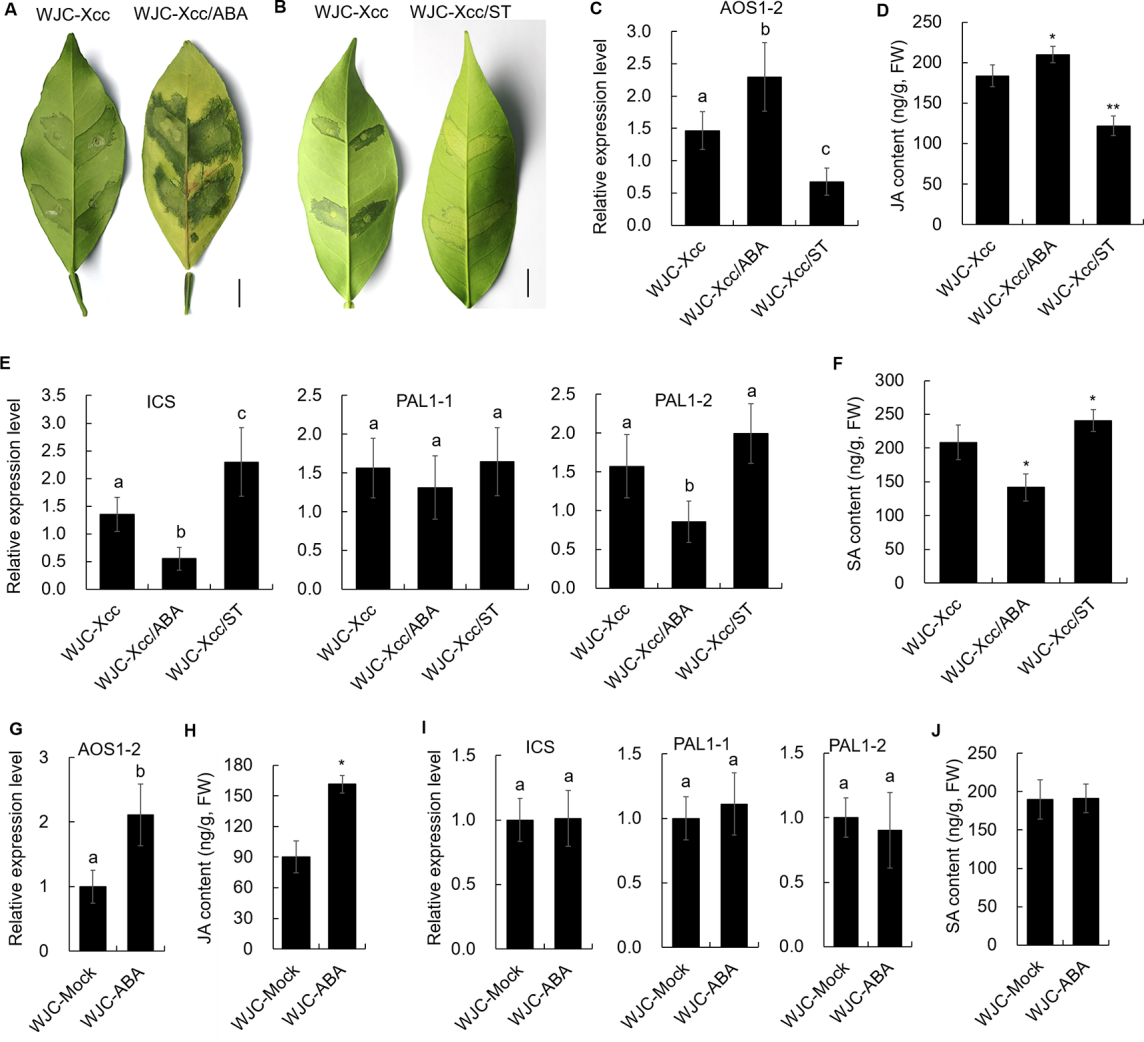
Effects of ABA and its synthesis inhibitor on canker resistance and JA and SA synthesis in Wanjincheng orange. **(A)** Effects of exogenous ABA on the canker resistance of Wanjincheng orange leaves infected with Xcc. **(B)** Effects of ABA synthesis inhibitor ST on the canker resistance of Wanjincheng orange leaves infected with Xcc. Photographs were taken at 6 days after inoculation. Scale bars = 1 cm. **(C)** Relative expression of the *AOS1-2* gene in Wanjincheng orange leaves induced by exogenous ABA after infection with Xcc. **(D)** JA content in citrus leaves treated with ABA after infection with Xcc. **(E)** Relative expression levels of genes involved in SA synthesis in Wanjincheng orange leaves induced by exogenous ABA after infection with Xcc. **(F)** SA content in citrus leaves treated with ABA after infection with Xcc. **(G)** Relative expression level of the *AOS1-2* gene in Wanjincheng orange after the ABA treatment. **(H)** JA content in Wanjincheng orange leaves after the ABA treatment. **(I)** Relative expression levels of genes involved in SA synthesis in Wanjincheng orange leaves induced by exogenous ABA. **(J)** SA content in citrus leaves treated with ABA. All the samples were detected at 3 days after treatment. Leaves treated with Xcc **(C**–**F)** or water **(G**–**J)** were used as control. Error bars of content determinations indicate the SDs of three biological repeats. Statistical significance related to the control was determined by two-tailed Student’s *t*-test (*, *P* < 0.05; **, *P* < 0.01). Error bars of expression levels represent the SDs of three biological replicates. Different letters above bars represent signiﬁcant differences from the control based on Duncan’s multiple range test (*P* < 0.05). WJC, Wanjincheng orange; ABA, abscisic acid; ST, sodium tungstate; AOS, 13-allene oxide synthase; JA, jasmonic acid; ICS, isochorismate synthase; PAL, phenylalanine ammonia lyase; SA, salicylic acid; FW, fresh weight.

To reveal the mechanism involved in Xcc’s regulation of JA signals to favor disease development in the Wanjincheng orange (S), we focused on the cis-regulating motifs in the 1.5-kb promoters of *AOS1-2* genes in the two cultivars, because this gene may play a crucial role in the accumulation of JA in response to Xcc (**Figure 3**). Strikingly, the core ABA-responsive elements (ABRE) motif (ACGTG) is overrepresented in the *AOS1-2* promoter of Wanjincheng orange (S) but absent in the *AOS1-2* promoter of Jindan (R). Specifically, there are five ABREs in the *AOS1-2* promoter of Wanjincheng orange (S) (**Figure 7A**). The different number of ABREs led us to investigate the correlation between ABA and JA in the process of Xcc infection. The ABA content increased in Wanjincheng orange (S) upon Xcc infection, but it showed no significant difference between mock and Xcc treatments in Jindan (R) (**Figure 7B**). qRT-PCR further revealed that genes involved in ABA-synthesis and -signaling pathways were specifically upregulated by Xcc in Wanjincheng orange (S) but not upregulated as much in Jindan (R) (**Figures 7C**, **D**). These results suggested that the increased ABA level may contribute to canker susceptibility in Wanjincheng orange (S). To confirm this hypothesis, we treated Wanjincheng orange leaves with ABA and sodium tungstate (ST, an ABA synthesis inhibitor). Exogenous ABA treatments reduced the basal disease resistance, while ST treatments increased the disease resistance in Wanjincheng orange (S) (**Figures 8A**, **B**), which itself has no adverse effects on bacterial viability (**Supplementary Table 2**). Furthermore, both the *AOS1-2* expression and JA levels were upregulated by the ABA treatment in Wanjincheng orange (S) during Xcc infection, while ST significantly repressed the Xcc-induced JA accumulation in Wanjincheng orange (**Figures 8C**, **D**). In contrast, the ABA treatment decreased SA synthesis, and ST increased SA synthesis in Wanjincheng orange (S) after Xcc infection (**Figures 8E**, **F**). Moreover, JA synthesis was induced, while SA accumulation was not affected, in Wanjincheng orange leaves only treated with ABA (**Figures 8G**–**J**). Thus, ABA may contribute to canker susceptibility by regulating JA synthesis in susceptible citrus.

## Discussion

Determining dynamic phytohormone concentrations and the resulting signals are direct and effective approaches to reveal the hormonal crosstalk that regulates plant immunity. In this study, we showed that JA biosynthesis is significantly induced upon Xcc infection and contributes to the disease susceptibility of citrus by counteracting the SA pathway. These results suggested that the principle of JA–SA antagonism also works in tree plants as it does in non-tree plants, such as *Arabidopsis* and rice (Thaler et al., 2012). A fundamental question is why different citrus genotypes respond in opposite fashions with regard to the SA–JA balance, with the SA and JA pathways being upregulated upon Xcc infection in the resistant and susceptible cultivars, respectively (**Figures 2** and **3**). Here, we showed that ABA is an important hormone that regulates JA–SA antagonism and affects the resistance of citrus to canker disease. We further found that citrus *AOS1-2*, encoding an enzyme that catalyzes the first committed step in the JA biosynthesis from hydroperoxides of α-linolenic acid (Mueller, 1997), was significantly upregulated in Wanjincheng orange (S), but not in Jindan (R), upon Xcc infection (**Figure 3B**). However, the homolog *AOS1-1* was significantly upregulated in both Wanjincheng orange (S) and Jindan (R) (**Figure 3B**), implying that *AOS1-1* was probably not responsible for the different JA biosynthesis patterns observed in the two citrus cultivars. Thus, *AOS1-2* was most likely responsible. To investigate the mechanism accounting for the different inducibility level of *AOS1-2* upon Xcc infection, we compared the promoter sequences of *AOS1-2* in the two cultivars and found that ABRE is overrepresented in the *AOS1-2* promoter of Wanjincheng orange (S) but absent in that of Jindan (R) (**Figure 7A**). The multiple ABRE motifs in the promoter of *AOS1-2* might contribute to the Xcc-induced JA biosynthesis and disease susceptibility. Consistently, ABA biosynthesis is induced in Wanjincheng orange (S) upon Xcc-treatment, but not in Jindan (R) (**Figures 7B**, **C**). Exogenous ABA promoted the disease susceptibility and also induced the expression of *AOS1-2* in Wanjincheng orange (S), while its synthesis inhibitor ST had the opposite effects (**Figures 8A**–**C**). Thus, the inducibility of *AOS1-2* by ABA in Wanjincheng orange (S) may have resulted in the high JA level and disease susceptibility.

Many pathogens hijack host ABA biosynthesis or produce ABA themselves to promote disease (Berens et al., 2017). The negative impact of ABA on SA-mediated defenses has been best studied in *Arabidopsis* and rice. In these plants, the resistance against pathogen is reduced when the ABA pathway is upregulated and increased when the ABA pathway is downregulated (De Torres‐Zabala et al., 2007; Xu et al., 2013; Xiao et al., 2017; Liao et al., 2018). Although the evidence for ABA–SA antagonism is increasing, the underlying mechanisms are not well understood (Cao et al., 2011; Lievens et al., 2017; Xiao et al., 2017). In this study, both ABA and JA biosynthesis were significantly induced by Xcc in Wanjincheng orange (S) but not in Jindan (R), providing a unique perspective. To our knowledge, this is only the second example in which both ABA and JA are hijacked by a bacterial pathogen to cause disease. The first example comes from the interaction between *Arabidopsis* and *Pseudomonas syringae* pv. *Tomato* (De Torres‐Zabala et al., 2007; Mine et al., 2017). As discussed above, ABA might induce JA biosynthesis through an ABA-responsive JA biosynthetic enzyme, which inspired us to propose the following hypothesis: ABA acts upstream of JA to antagonize SA. This hypothesis is supported by ABA being required for JA biosynthesis and JA-induced defenses against *Pythium irregulare*, a necrotrophic oomycete pathogen, in *Arabidopsis* (Adie et al., 2007). Additionally, a mutant overexpressing an ABA biosynthetic enzyme-encoding gene accumulates more ABA and JA, but less SA (Fan et al., 2009). Furthermore, both ABA and JA pathways contribute to pathogen virulence. *Arabidopsis* overcomes the virulence by blocking JA-signaling activation, suggesting that JA may act downstream of ABA (Mine et al., 2017). Thus, it appears that ABA acts upstream of JA to restrict necrotrophic pathogens and this antagonizes SA, which restricts biotrophic pathogens. Further genetic experiments are needed to confirm the hypothesis.

The conventional genetic improvement of citrus is restrained by the long juvenile period, pollen incompatibility, parthenocarpy, and polyembryony (Talon and Gmitter, 2008). Recently, the genome-editing technique, CRISPR-Cas, has shown promise for disease-resistance breeding in citrus (Jia et al., 2017; Jia et al., 2019b). Susceptibility genes (*S* genes) that promote pathogen infection are prime targets for genome editing to improve disease resistance (Schie and Takken, 2014; Langner et al., 2018). For citrus canker, this *S* gene editing-mediated disease-resistance breeding is especially important and promising, as no resistance genes corresponding with Xcc have been identified in citrus or citrus-related species (Ference et al., 2018). However, the information on *S* genes is only one prerequisite for genome editing and is minor in the term of Xcc–citrus interactions. Currently, only one *S* gene, *LATERAL ORGAN BOUNDARIES 1*, has been identified as being induced by Xcc (Hu et al., 2014). The cis-motifs in this gene’s promoter, which are responsible for the Xcc-induction, can be edited, resulting in the resistance against Xcc being successfully improved (Peng et al., 2017; Jia et al., 2019a). Identifications of new *S* genes are helpful for the further improvement of citrus canker resistance. Our study suggests that *AOS1-2* in Wanjincheng orange (S) is a candidate *S* gene. More importantly, editing the multiple ABREs within the *AOS1-2* promoter of Wanjincheng orange (S) has the promise of altering the adverse hormonal crosstalk and to improve citrus canker resistance without interrupting normal hormonal crosstalk and plant fitness.

**Figure 9:**
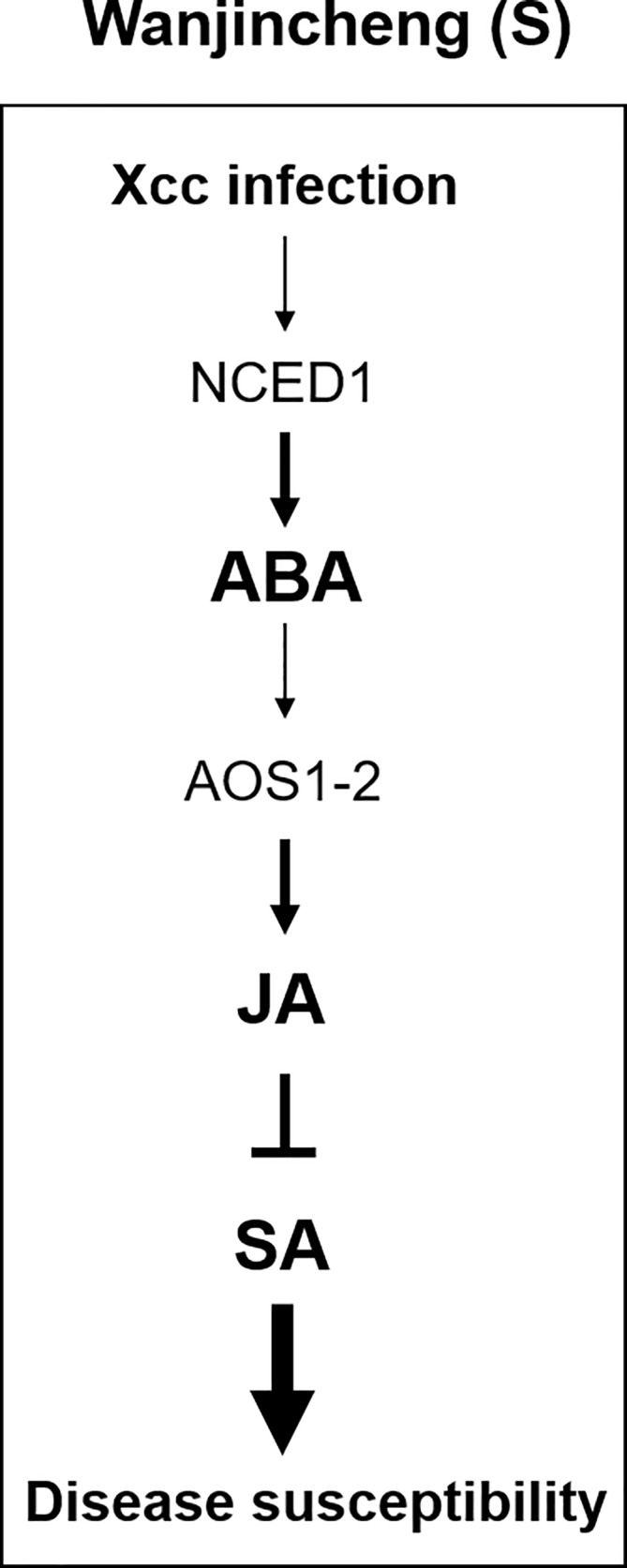
Model for the interplay among JA, SA, and ABA in Wanjincheng orange (S) during infection with Xcc. The infection of the leaves with Xcc leads to an increased ABA accumulation through NCED1 in Wanjincheng orange (S). The high ABA content in susceptible citrus promotes JA synthesis through the ABA-responsive AOS1-2, which in turn antagonizes the effectual SA-mediated defenses to promote canker disease susceptibility.

Thus, we propose a model to explain how Xcc bacteria manipulate the hormonal balance of susceptible citrus to establish compatible interactions. Xcc infection leads to the induction of ABA biosynthesis, and ABA promotes JA biosynthesis through the enhanced expression of ABA-responsive *AOS1-2* in Wanjincheng orange (S). This, in turn, antagonizes the effectual SA-mediated defenses to promote disease (**Figure 9**). Our findings provide new insights into the molecular mechanisms underlying canker-resistant and -susceptible citrus cultivars and suggests a new target gene for the biotechnological improvement of citrus canker resistance.

## Data Availability Statement

Publicly available datasets were analyzed in this study. This data can be found here: http://bioinformatics.psb.ugent.be/webtools/plantcare/html/.

## Author Contributions

SC, XZ, and QLo designed experiments. QLo, YX, YH, and QLi performed experiments. QLo and YX analyzed the data. QLo wrote the manuscript. SC supervised the research.

## Funding

This research was supported by the National Key Research and Development Program of China (2018YFD1000300 to SC), the Fundamental Research Funds for the Central Universities (XDJK2019C027 to QLo) and the Earmarked Fund for China Agriculture Research System (CARS-26 to SC).

## Conflict of Interest

The authors declare that the research was conducted in the absence of any commercial or financial relationships that could be construed as a potential conflict of interest.
