# Supplementary material for: Abscisic Acid Promotes Jasmonic Acid Accumulation and Plays a Key Role in Citrus Canker Development
Source: Front Plant Sci. 2019 Dec 20;10:1634. doi: 10.3389/fpls.2019.01634 (PMC6934002; doi:10.3389/fpls.2019.01634)
Supplement: Supplementary file 1 [file Table_1.docx]

**Supplementary Table 1** Primers used for qRT-PCR

| Gene ID | Primer name | Primer sequence (5'to3') | Annealing temperature (°C) | Product length (bp) |
| --- | --- | --- | --- | --- |
| Cs1g05000 | Actin-F | CTGCCTGATGGCCAGATCAT | 60 | 97 |
|  | Actin-R | TACCAGCAGCTTCCATTCCG |  |  |
| Cs7g24940 | PAL1-1-F | GGTTTAGCTGCCACGGTACT | 60 | 146 |
|  | PAL1-1-R | ATTTGTCCCGGATGGTGCTT |  |  |
| Cs6g11950 | PAL1-2-F | CAACGCCCAAGTCAAAGTGG | 60 | 119 |
|  | PAL1-2-R | AACCAGTGGTAACGCCGTAG |  |  |
| Cs5g04210 | ICS-F | TTGGCGGGCAGACTAAGAAG | 60 | 152 |
|  | ICS-R | ACTGGCCCAGCATACATTCC |  |  |
| orange1.1t04376 | LOX2-F | CCACGGTGTGACACAGATCA | 60 | 165 |
|  | LOX2-R | AGGATCGACAAAGGCGGTTT |  |  |
| Cs5g28310 | AOS1-1-F | CAACATGCCTCCTGGTCCTT | 60 | 123 |
|  | AOS1-1-R | GTGCCAACAAAAACGTTGCG |  |  |
| Cs3g24230 | AOS1-2-F | CCACCTACACGGAGGCATTT | 60 | 129 |
|  | AOS1-2-R | CGGGGTTCTTACCGAACCAA |  |  |
| orange1.1t03729 | OPR3-F | ACAGGACCGATCAATACGGC | 60 | 167 |
|  | OPR3-R | GAGGCCAAGTGCTTCAGGAT |  |  |
| Cs1g02310 | TGA1-F | AAGCCAGGCTGACCATCTTC | 60 | 97 |
|  | TGA1-R | CTCACCCAAAGCAAGCAACC |  |  |
| Cs8g15030 | TGA2-F | TCCGGCGGATATTGACAGTG | 60 | 100 |
|  | TGA2-R | CGAGATGCCCAAAGGGAACT |  |  |
| Cs4g14600 | NPR1-F | GGGTTTGATCCGCTTGTTGC | 60 | 86 |
|  | NPR1-R | TCCACACAAACACAAACGCC |  |  |
| Cs2g10790 | NPR3-F | TGGAGGTGTCAACTTGCGTT | 60 | 121 |
|  | NPR3-R | AGTGAAACCAACTCCGGCAA |  |  |
| Cs8g03430 | PR1-1-F | CGTTTGGCGCAAATCTGTGA | 60 | 135 |
|  | PR1-1-R | GGTGAGTCTTGTGCATGGGA |  |  |
| Cs7g29570 | WRKY70-F | AAGATGGGTTGGTTTCGGCT | 60 | 142 |
|  | WRKY70-R | CAAGAATTCGCCTGCTGCTG |  |  |
| Cs7g31600 | COI1-1-F | CTGACGAGGGGCTTATAGCG | 60 | 92 |
|  | COI1-1-R | TGCAAGTGCATACTCGCTGA |  |  |
| Cs5g32500 | COI1-2-F | TTGCTGCCGATTGCAAGAAC | 60 | 187 |
|  | COI1-2-R | AGATTGGGACACCGAACCAC |  |  |
| orange1.1t00550 | MYC2-1-F | GGGCTCAAAGCTCTGGTTCT | 60 | 97 |
|  | MYC2-1-R | ATGCCATGCGATTGTGCTTC |  |  |
| orange1.1t01021 | MYC2-2-F | TGACGGACTACCGGTTACCT | 60 | 111 |
|  | MYC2-2-R | ACTGCGAAGGTGGCCAAATA |  |  |
| Cs2g03270 | NCED1-1-F | TGGGATGGTGAATCGCAACA | 60 | 161 |
|  | NCED1-1-R | ATGGCTCGCCGCCATATTTA |  |  |
| Cs5g14370 | NCED1-2-F | TATGACTCCTGCCGACTCCA | 60 | 191 |
|  | NCED1-2-R | AGTACGCGAAACGGGTCTTT |  |  |
| Cs8g19140 | PP2C-3-F | AGCTTCACGCGTATGGACAA | 60 | 114 |
|  | PP2C-3-R | AACAGCAGTAGACCCAACGG |  |  |
| Cs9g18020 | PP2C-8-F | TGCTAGCGATGGCTTATGGG | 60 | 80 |
|  | PP2C-8-R | TTATCCGGCCGCTGAAACAT |  |  |
