# Supplementary material for: Abscisic Acid Promotes Jasmonic Acid Accumulation and Plays a Key Role in Citrus Canker Development
Source: Front Plant Sci. 2019 Dec 20;10:1634. doi: 10.3389/fpls.2019.01634 (PMC6934002; doi:10.3389/fpls.2019.01634)
Supplement: Supplementary file 2 [file Table_2.docx]

**Supplementary Table 2** Effect of ST on bacterial viability

| Treatment | 4h | 8h |
| --- | --- | --- |
| Xcc | 0.605±0.008 | 1.156±0.033 |
| Xcc+ST | 0.614±0.011 | 1.131±0.032 |

Xcc suspensions of the same concentration and volume were cultured with or without ST (5-mM), then OD600 values were detected at 4h and 8h, respectively. Error bars indicate the SDs of three biological repeats. Statistical significance related to the Xcc control was determined by two-tailed Student’s *t*-test (P > 0.05). Xcc, *Xanthomonas citri* ssp. *citri*; ST, sodium tungstate.
